# Thoracic esophageal injury due to a neck stab wound: a case report

**DOI:** 10.1186/s40792-021-01208-5

**Published:** 2021-05-20

**Authors:** Masaki Honda, Toshiro Tanioka, Shigeo Haruki, Yuko Kamata, Hiromasa Hoshi, Kyoko Ryu, Kenta Yagi, Kodai Ueno, Satoshi Matsui, Yoshiteru Ohata, Fumi Hasegawa, Akio Kaito, Kaida Arita, Koji Ito, Noriaki Takiguchi

**Affiliations:** 1 Department of Digestive Surgery, Tsuchiura Kyodo General Hospital, 4-1-1 Otuno, Tsuchiura-shi, Ibaraki, Japan; 2 Department of Esophageal Surgery, Tokyo Metropolitan Cancer and Infectious Diseases Center Komagome Hospital, 3-18-22 Hokomagome, Bunkyo, Tokyo, Japan

**Keywords:** Esophageal injury, Esophageal perforation, Thoracic esophagus, Neck trauma, Penetrating, Stab wound

## Abstract

**Background:**

Traumatic esophageal injury leads to severe complications such as mediastinitis, pyothorax, and tracheoesophageal fistula. Although prompt diagnosis and treatment are required, there are no established protocols to guide diagnosis or treatment. In particular, thoracic esophageal injury tends to be diagnosed later than cervical esophageal injury because it has few specific symptoms. We report a case of thoracic esophageal injury caused by a cervical stab wound; the patient was stabbed with a sharp blade.

**Case presentation:**

A 74-year-old woman was attacked with a knife while sleeping at home. The patient was taken to the emergency room with an injury localized to the left section of her neck. She was suspected of a left jugular vein and recurrent laryngeal nerve injury from cervical hematoma and hoarseness. On the day following the injury, computed tomography revealed a thoracic esophageal injury. Emergency surgery was performed for an esophageal perforation and mediastinal abscesses. Although delayed diagnosis resulted in suture failure, the patient was able to resume oral intake of food a month later following enteral feeding with a gastrostomy. Esophageal injuries due to sharp trauma are rare, and most are cervical esophageal injuries. There are very few reports on thoracic esophageal injuries.

**Conclusions:**

The possibility of thoracic esophageal injury should always be considered when dealing with neck stab wounds, particularly those caused by an attack.

## Background

A traumatic esophageal injury is a rare condition [1]. Although a sore throat, minor subcutaneous emphysema, and hematemesis are esophageal injury symptoms, they are also found with cervical trauma [2]. Due to this, it is difficult to diagnose esophageal injury based on these symptoms only. Moreover, thoracic esophageal injury has no specific symptoms, and early diagnosis tends to be challenging and delayed [1, 3]. We encountered a surgical case of thoracic esophageal injury caused by a cervical stab wound which was detected late due to a sharp blade injury.

## Case presentation

Patient: 74-year-old female.

Chief complaint: stab wound to the neck.

Medical history, family history: none to be mentioned.

Present medical history: when waking up to go to the bathroom around 2:00 a.m., the patient was stabbed in the neck by an unknown person. At the time of transport, her pulse rate was 60 beats/min and systolic blood pressure was 80 mmHg. A blood transfusion stabilized her vital signs after arriving at the hospital. A computed tomography (CT) scan revealed disruption of the internal jugular vein and bleeding from the same vessel, and the patient was admitted to our hospital. The weapon was unknown at the time of admission.

Vitals on arrival: GCS E4V5M6, body temperature 37.4 °C, pulse 66 beats/min, blood pressure 159/69 mmHg, respiratory rate 14 breaths/min, and SpO_2_ 100% (room air).

Physical examination: cut wounds were found on the left of the patient's neck, left shoulder, right anterior chest, and right forearm. The left cervical region was the deepest wound, and hemostasis with blood clots was found. No apparent air leak was observed. Difficulty in opening the left eye and hoarseness were observed.

### CT findings (Fig. 1)

**Fig. 1:**
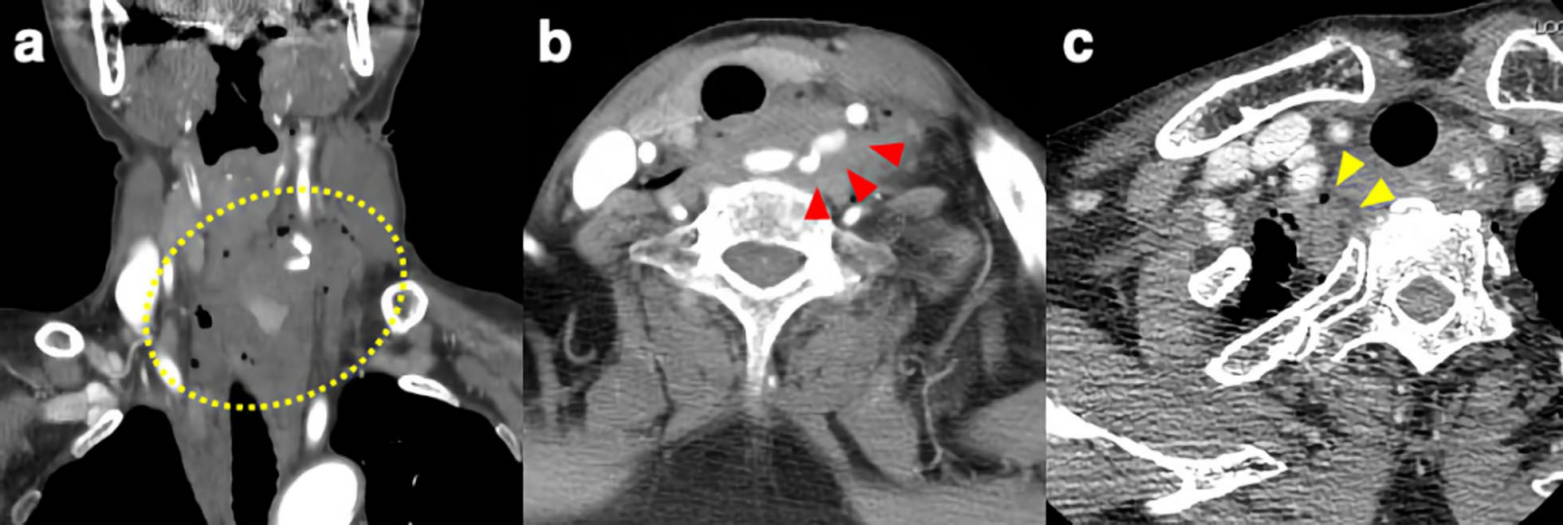
CT at the time of transport to the hospital. a The hematoma was spread from the left neck to the right upper mediastinum (yellow dotted line). b The internal jugular vein was damaged, and extravascular leakage from the vein was observed (red arrowheads). c There was pulmonary contusion in the right upper lobe but no hemopneumothorax (yellow arrowheads)

The hematoma spread from the left side of the neck to the right upper mediastinum. The internal jugular vein was damaged, and extravascular leakage from the vein was observed. There was pulmonary contusion in the right upper lobe, but no hemopneumothorax.

### Treatment plan

**Fig. 2:**
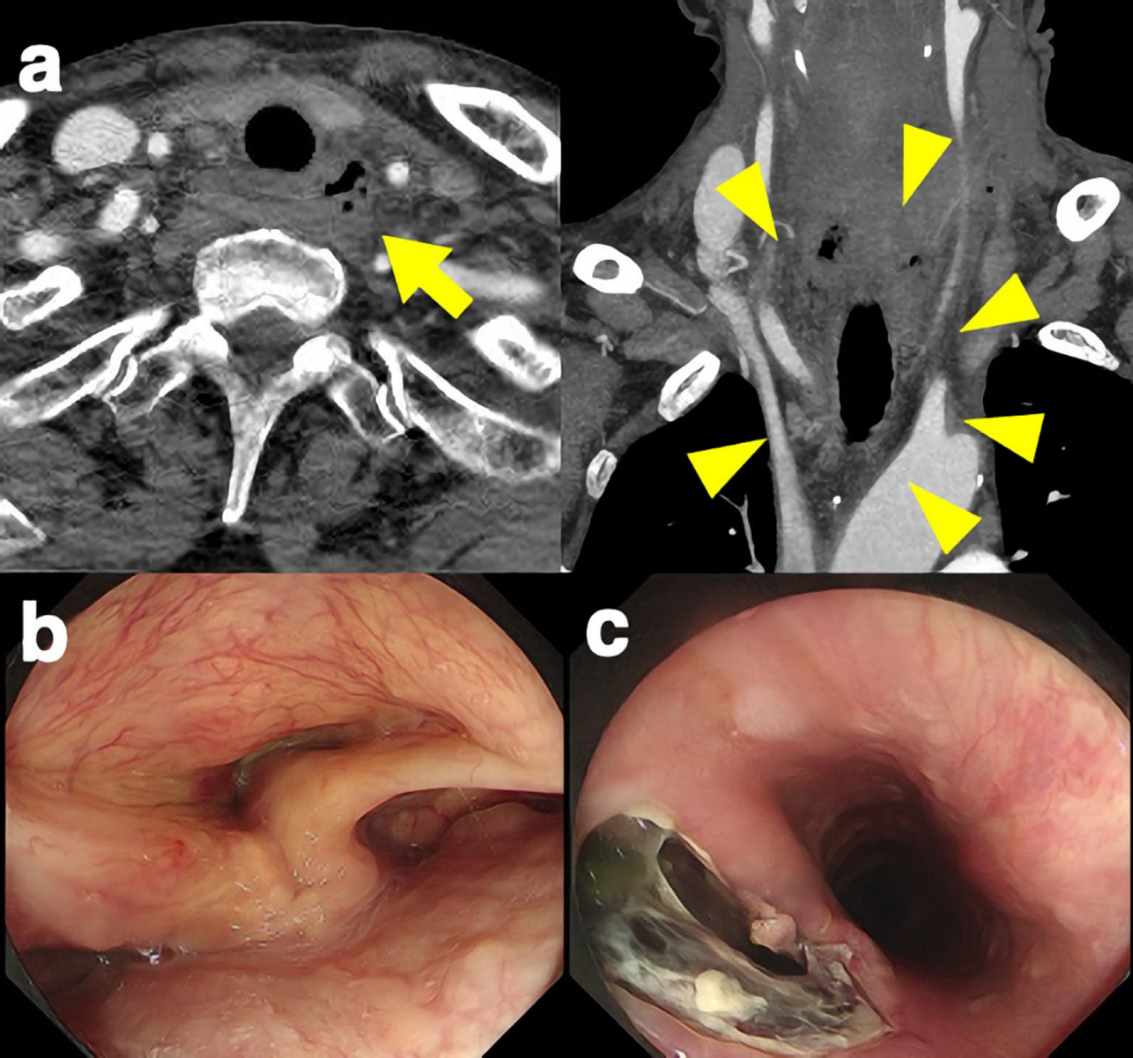
Examinations on the second day after the injury. CT revealed free gas (yellow arrow) and fluid accumulation (yellow arrowheads) around the thoracic esophagus (**a**). Upper gastrointestinal endoscopy shows left vocal cord palsy (**b**) and injury site on the esophageal wall (**c**)

The neck surgeons performed an emergency operation. After opening the neck wound, damage to the left internal jugular vein and left vagus nerve were observed, and they were ligated. There was no recurrent laryngeal nerve injury. A hematoma of the right upper mediastinum was observed. The patient was started on food intake the following day. On the second day after the injury, a follow-up CT scan revealed free gas and fluid accumulation around the thoracic esophagus (Fig. 2a), and an esophageal injury was suggested.

### Upper gastrointestinal endoscopy (Fig. 2b, c)

An endoscopy revealed a left vocal cord palsy. An injury site was found on the left side of the posterior wall of the esophagus, 20 cm from the incisor.

The patient was diagnosed with esophageal injury and underwent emergency surgery.

### Intraoperative findings (Fig. 3)

**Fig. 3:**
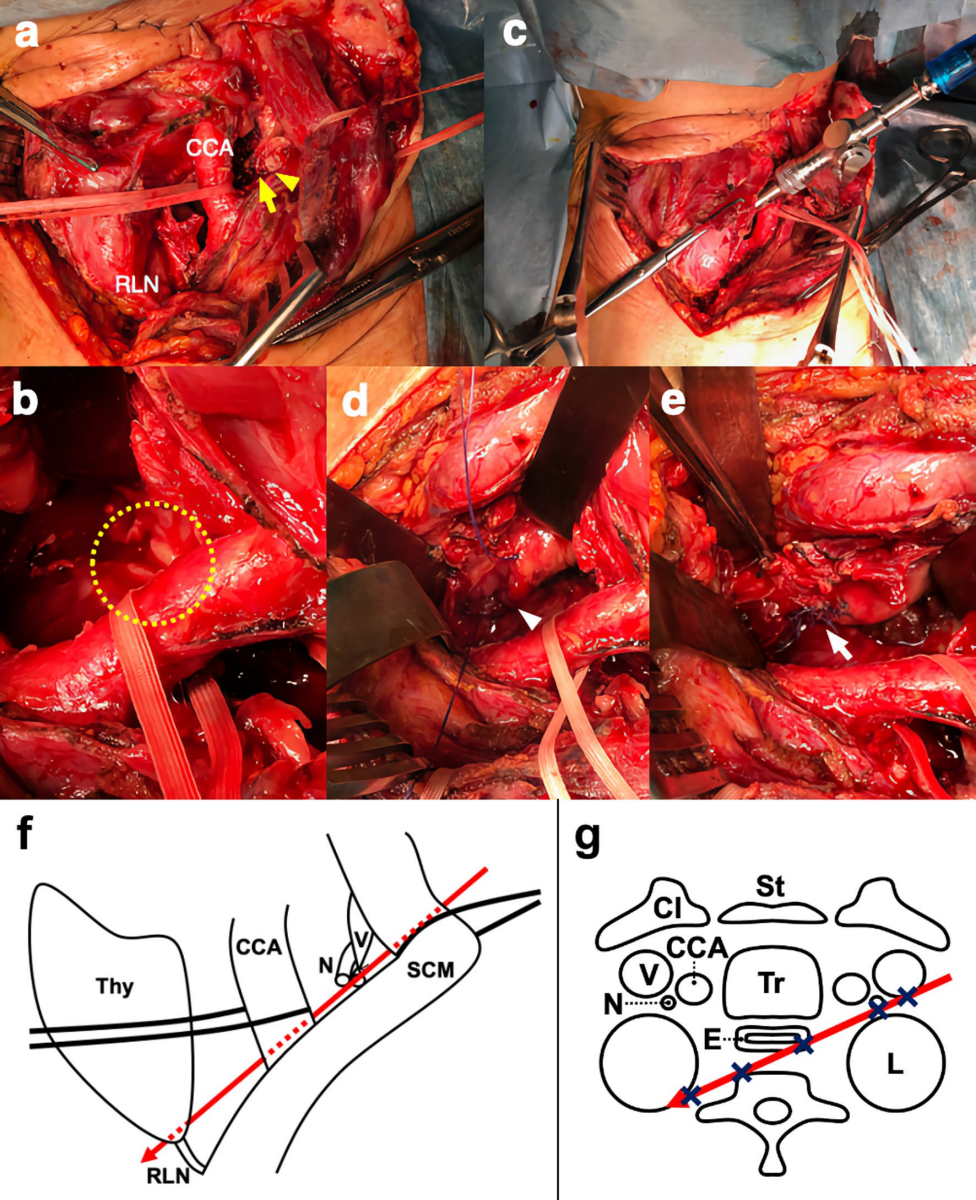
Intraoperative findings. a The common carotid artery and the damaged vagus nerve (yellow arrow) were found on the ligated internal jugular vein’s dorsal side (yellow arrowhead). CCA: common carotid artery. RLN: recurrent laryngeal nerve. b A scar was found on the anterior surface of the vertebral body (yellow dotted line). c The scar reached the right thoracic cavity. d A 2-cm injury site on the left esophageal wall near the sternum's superior border was present (white arrowhead). e The injury site was sutured using absorbable thread (white arrow). f The scheme of intraoperative viewing. g The scheme of the horizontal cross section. Red arrow is wound pathway and x is the damaged part. *Thy* thyroid gland. *CCA* common carotid artery. *N* vagus nerve. *V* internal jugular vein. *RLN* recurrent laryngeal nerve. *SCM* sternocleidomastoid. *Cl* clavicle. *St* sternum. *Tr* trachea. *E* esophagus. *L* lung

An endoscopic gastrostomy was performed before neck surgery. The neck wound was reopened, and the ligated internal jugular vein was observed. The common carotid artery and damaged vagus nerve were found on the dorsal side of the vein (Fig. 3a). The anterior vertebral lobe was observed between the common carotid artery and the esophagus. After thorough cleaning of the surrounding hematoma, a scar was found on the anterior surface of the vertebral body, which appeared to have been cut by a weapon (Fig. 3b). The vein on the anterior surface of the vertebral body was torn, and bleeding was stopped with a clip. The scar reached the right thoracic cavity (Fig. 3c), but there was no apparent damage to the right lung or other organs. Careful inspection of the esophageal wall revealed a 2-cm injury site on the left esophageal wall near the superior border of the sternum (Fig. 3d). The injury site was sutured using absorbable thread (Fig. 3e). After confirming no air leakage or stenosis of the lumen with endoscopy, drains were placed into the right thoracic cavity along the stab wound from the left neck to evaluate hemothorax and pneumothorax and beside the esophagus to assess the presence of suture failure, respectively, and the operation was completed.

**Fig. 4:**
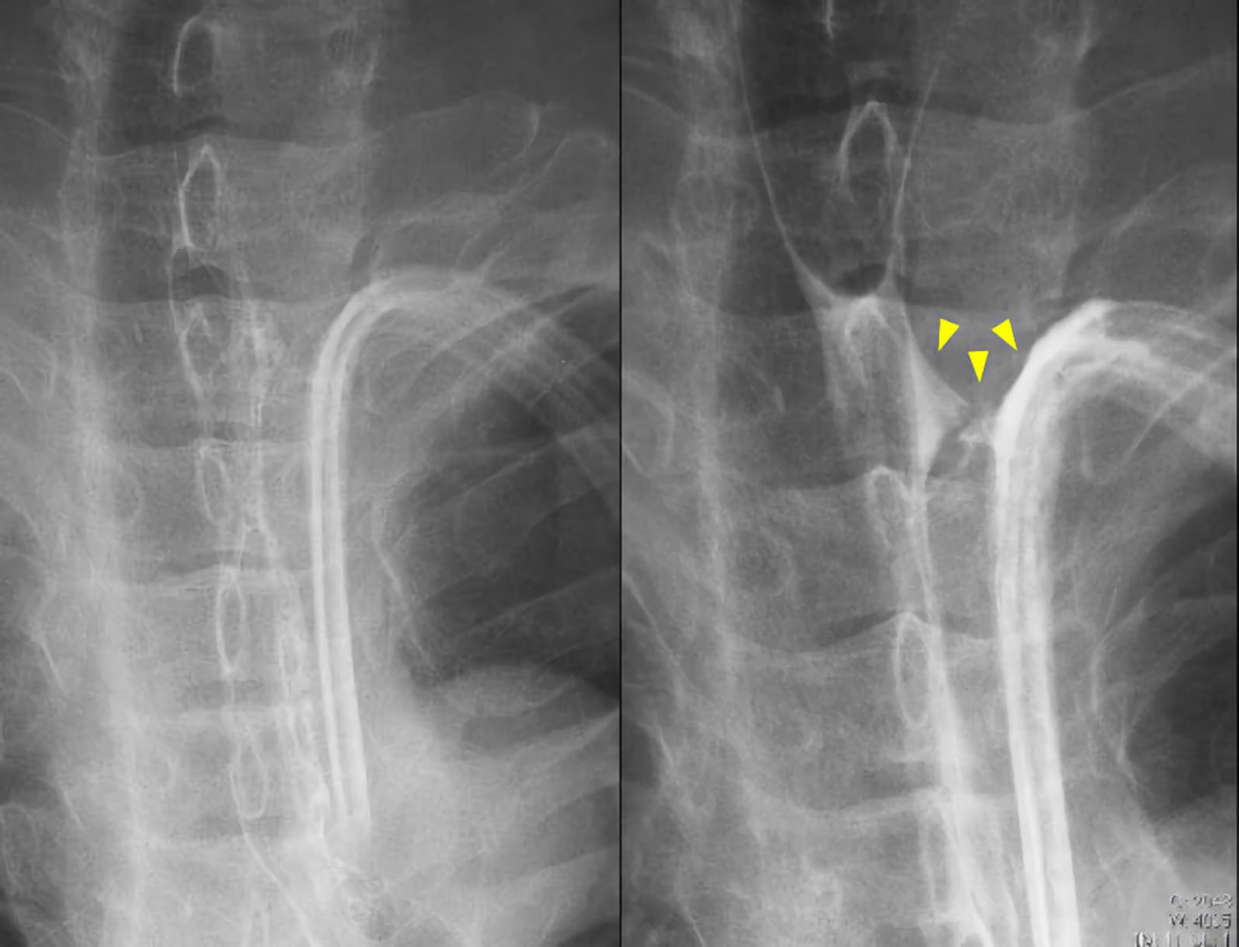
Esophagogram on the ninth postoperative day. Contrast leakage (yellow arrowheads)

**Fig. 5:**
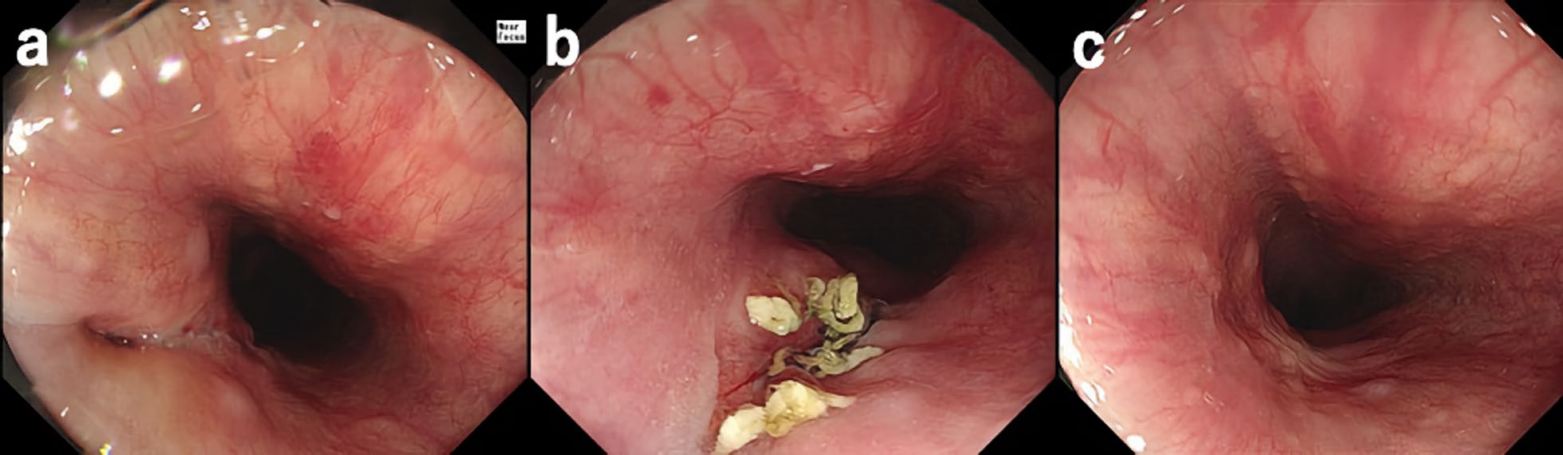
Postoperative endoscopy. **a** Two weeks after the surgery. **b** A month after the surgery. **c** Three months after the surgery

Postoperative course: the patient started drinking water on the fourth postoperative day, and there was no problem with the drain characteristics. The right thoracic drain was removed on the sixth postoperative day with no findings suspicious for hemothorax or pneumothorax. However, on the ninth postoperative day, an esophagogram showed contrast leakage into the esophagus drain (Fig. 4), and a suture failure was revealed. Gastrostomy feeding was started on postoperative day 10, and albumin improved from 3.1 g/dl (before leakage) to 3.5 g/dl (before oral feeding). The fistula was treated for a month since she also had dysphagia associated with recurrent nerve palsy. Oral intake was initiated, and she was transferred to another hospital for rehabilitation. Three months after the surgery, endoscopy revealed a scar at the injury site (Fig. 5).

## Discussion

There are few comprehensive reports of neck trauma. Injury to major organs, such as the common carotid artery, internal jugular vein, pharynx, trachea, and esophagus, results in a fatal outcome. There is not a standard treatment protocol for neck stab wounds [4]. According to a report by Demetriades, esophageal injury was observed in 0.9% of penetrating neck injuries and commonly caused by gunshot wounds (0.5%), followed by stab wounds (0.3%) [2]. In Japan, gunshot wounds are uncommon, therefore the majority of neck injuries are stab wounds caused by knives, and most are suicide attempts [5]. It has been reported that 90% of neck stab wounds involve the shallow cervical region, which is shallower than the vast lateral, sternocleidomastoid, and anterior cervical muscle groups, and that deep neck injuries are rare [6]. Previous reports suggest that esophageal injuries, especially those involving the thoracic esophagus rather than the cervical esophagus, have a high mortality rate [1, 7]. Therefore, esophageal injuries should not be overlooked.

Zone classification has long been used to diagnose neck injuries [8, 9]. They are divided into three zones, from the clavicle to the base of the skull. In Zone II (from the level of the cricoid cartilage to the mandibular angle), which accounts for the majority of neck injuries, there are many vital organs including the common carotid artery, internal jugular vein, trachea, and esophagus. It has been reported that more than half of patients with significant cervical vessel injuries die [10].

Esophageal injury may cause a sore throat, minor subcutaneous emphysema of the neck, and hematemesis. However, these are common symptoms of neck trauma and are not specific to esophageal injuries [2]. In particular, perforation of the thoracic esophagus rarely presents with specific symptoms, and diagnosis tends to be delayed in the upper and middle thoracic esophagus compared to the cervical and lower thoracic esophagus [3]. Although conservative treatment may be an option for esophageal perforation depending on the condition, the longer the time since the onset of the disease, the more likely the perforation is to be necrotic and fragile due to high contamination. These patients require surgical treatment [11]. In idiopathic rupture of the esophagus, Cameron et al. reported that conservative treatment (such as thoracic drainage and intermittent continuous suctioning of the esophagus) is possible when all the following conditions are met: (i) rupture is confined to the mediastinum; (ii) drainage into the esophagus through the rupture site; (iii) symptoms are mild, and (iv) there is no severe infection [12].

On the other hand, Zenga et al. reported that surgical treatment is necessary when any of the following conditions are met: (i) oral intake from injury to diagnosis; (ii) passage of more than 24 h from injury to diagnosis, and (iii) deterioration of general condition [13]. In addition, the longer the time since the onset of the injury, the more likely it is that suture failure will occur after closure, and Anderson states that 24 h after onset is the safe limit for direct suture closure without suture failure [14]. Considering these facts, we assumed a high risk of suture failure in upper mid-thoracic esophageal injuries. Our case also fulfilled Zenga's requirements (i) and (ii), and surgical treatment was appropriate. We attempted direct suture closure because of mild contamination, lack of severe infection, and relatively small perforation diameter, and performed gastrostomy in anticipation of suture failure. Unfortunately, postoperative suture failure occurred, but the patient could recover by using an external fistula with a drain and nutritional therapy using a gastrostomy. There are some reports of delayed diagnosis and difficult suture closure, esophagectomy, cervical esophageal fistula and gastrostomy, and two-stage reconstruction may be performed [3, 15].

While angiography, CT, esophagography, and endoscopy are recommended to diagnose esophageal injury, evaluation is often difficult [16]. The injury mechanism also aids in the diagnosis of an injured organ. In the case of self-injury, the blade tip is thought to enter the wound level with or more cranial to the skin wound. Conversely, when another person swings the blade, the blade tip is likely to enter caudal to the skin wound. Therefore, it is necessary to assume the possibility of organ damage caudal to the cutaneous wound when harming others. In this case, there was an esophageal injury caudal to the neck wound. Some studies argue against using the zone classification approach, stating that there is no correlation between the height of the trauma site and the internal injury site [17].

In our case, we could diagnose esophageal injury at a relatively early stage, and the patient did not develop serious complications such as mediastinitis, pyothorax, or tracheoesophageal fistula. Considering that it was a neck stab wound caused by the attack, the weapon was unknown, and the CT findings showed a right upper lobe pulmonary contusion, we potentially could have diagnosed the esophageal injury earlier. The possibility of thoracic esophageal injury should always be considered when dealing with neck stab wounds caused by attackers.

## Conclusions

We report a case of thoracic esophageal injury caused by a neck stab wound. Although esophageal injury is challenging to diagnose because of the lack of specific clinical symptoms, it is advisable to always consider the possibility of thoracic esophageal injury in diagnosis and treatment, taking into account the path of the wound and CT findings.

## Data Availability

The datasets supporting the conclusions of this article are included within the article and its additional files.

## Abbreviations

CCA: Common carotid artery
Cl: Clavicle
CT: Computed tomography
E: Esophagus
L: Lung
RLN: Recurrent laryngeal nerve
SCM: Sternocleidomastoid
St: Sternum
Thy: Thyroid gland
Tr: Trachea
